# Follistatin Is Associated with Bone Mineral Density in Lean Adolescent Girls with Increased Physical Activity

**DOI:** 10.3390/children10071226

**Published:** 2023-07-14

**Authors:** Jaak Jürimäe, Liina Remmel, Anna-Liisa Tamm, Priit Purge, Katre Maasalu, Vallo Tillmann

**Affiliations:** 1 Institute of Sport Sciences and Physiotherapy, Faculty of Medicine, University of Tartu, 51008 Tartu, Estonia; liina.remmel@ut.ee (L.R.); priit.purge@ut.ee (P.P.); 2 Department of Physiotherapy and Environmental Health, Tartu Health Care College, 50411 Tartu, Estonia; annaliisatamm@nooruse.ee; 3 Institute of Clinical Medicine, Faculty of Medicine, University of Tartu, 50406 Tartu, Estonia; katre.maasalu@kliinikum.ee (K.M.); vallo.tillmann@kliinikum.ee (V.T.)

**Keywords:** follistatin, muscle, bone, energy expenditure, adolescent girls

## Abstract

Follistatin is a member of the activin–follistatin–inhibin hormonal system and is proposed to affect bone metabolism. However, data regarding the effect of follistatin on bone are relatively scarce and contradictory in humans. The purpose of the current study was to investigate possible associations of serum follistatin concentration with bone mineral characteristics in lean and physically active adolescent girls. Bone mineral density, body composition, resting energy expenditure and different energy homeostasis hormones in serum including follistatin, leptin and insulin were investigated. Significant relationships (*p* < 0.05) between serum follistatin (1275.1 ± 263.1 pg/mL) and whole-body (WB) bone mineral content (r = 0.33), WB areal bone mineral density (aBMD) (r = 0.23) and lumbar spine (LS) aBMD (r = 0.29) values were observed. Serum follistatin remained associated with LS aBMD independent of body fat and lean masses (r = 0.21; *p* < 0.05). However, the follistatin concentration explained only 3% (R^2^ × 100; *p* = 0.049) of the total variance in LS aBMD values. In conclusion, serum follistatin concentrations were associated with bone mineral values in lean adolescent girls with increased physical activity. Follistatin was an independent predictor of lumbar spine areal bone mineral density, which predominantly consists of trabecular bone.

## 1. Introduction

Adolescence is a period of rapid bone mineral accrual and is an important time to increase peak bone mass [1,2]. Mechanical factors, such as physical activity [2,3] and body composition [4,5], influence bone development during growth and maturation. Body composition variables including body fat mass (FM) and lean body mass (LBM) play an important role in bone mineral accrual during adolescence [6,7]. However, LBM as a measure of muscle mass rather than body FM is better correlated with areal bone mineral density (aBMD) in healthy adolescent and young females [7,8], and region-specific LBM has been suggested to be the strongest body composition determinant of aBMD in physically active adolescents [4,5]. In addition, muscle and bone tissue are biochemically and metabolically interrelated [9,10]. Specifically, muscle tissue can be classified as an endocrine organ that regulates bone metabolism through the synthesis and release of various myokines [11]. These myokines may have endocrine and paracrine effects on bone metabolism [9,10]. Among the different myokines identified, myostatin has been reported to be the most extensively studied myokine regarding its deleterious effect on bone remodeling [11]. Myostatin acts as a negative regulator of muscle mass and bone metabolism [12], while the inhibition of the myostatin pathway leads to muscle growth and an increase in bone mass [13]. The loss of muscle mass is also linked to low serum vitamin D levels [14,15], which may be due to the direct inhibition of myostatin expression by vitamin D [16,17]. On the other hand, it has also been suggested that myostatin possibly affects vitamin D metabolism via the regulation of fibroblast growth factor 23, which is a paracrine and endocrine mediator produced by bone cells [18]. However, it is well known that vitamin D is an important factor in bone health [2,19], although inconsistent results between vitamin D status and aBMD indices have been found in adolescent athletes due to their relatively high aBMD values for their age, which are the result of their specific training activity patterns [19,20]. In addition, follistatin is a myostatin-binding glycoprotein that antagonizes myostatin-induced inhibition of myogenesis and increases bone mineralization [21]. In contrast to studies on animal models, investigations regarding myokine effects on bone tissue are relatively scarce and contradictory in different human populations, especially during growth and maturation [10,11].

Follistatin is a myokine in the activin–follistatin–inhibin hormonal axis [22,23,24] that appears to regulate muscle growth through the inhibition of activin and myostatin [11,25]. Serum follistatin concentrations are increased in females with hypothalamic amenorrhea [24], and follistatin is affected by acute [26] and chronic [10,24,27] energy deficiencies in young women. Follistatin exerts metabolic benefits by reducing adiposity and improving glucose metabolism [28,29]. Follistatin levels have been found to be related to measures of FM and LBM in women with different body composition variables [9,29]. In addition to muscle mass, follistatin has been proposed to modulate bone mass by suppressing activin and myostatin signaling in bone cells [30]. It has been found that follistatin concentrations are related to bone density and volume, and to trabecular number and thickness in animal models [11,30,31]. Recently, circulating follistatin did not appear as an explicative independent variable of measured trabecular and cortical bone density in young women with different energy homeostasis states [10]. However, to the best of our knowledge, no investigations have been conducted to examine the association between follistatin concentration and bone mineral values in young women with chronic increases in energy expenditure. Accordingly, the purpose of the present study was to investigate the possible relationships between serum follistatin concentration and bone mineral indices in a homogeneous group of healthy, lean adolescent women with elevated energy expenditure.

## 2. Materials and Methods

### 2.1. Participants

Eighty-nine 14–18-year-old females (16.2 ± 1.3 years; 167.6 ± 5.0 cm; 58.1 ± 6.7 kg; body fat%: 24.1 ± 6.7%) with different physical activity levels took part in this investigation. All studied adolescent females were healthy without any injuries or diseases and were not taking any medication according to the health history questionnaire. Information about age at menarche, possible changes in the menstrual cycle, and vitamin or mineral supplementation was also collected [32]. The studied adolescent females consumed their everyday diet and were asked to remain on their ordinary everyday dietary intake [6]. All adolescent females were postmenarcheal; among them, 69 were eumenorrheic, 9 were oligomenorrheic and 11 had secondary amenorrhea. Eumenorrheic adolescent females were defined as regularly menstruating over the past 12 months, with menses occurring every 24–35 days [32,33]. Amenorrheic adolescent females failed to menstruate for at least 3 consecutive months after the initiation of menses and were not regularly menstruating for at least 6 months [33]. Oligomenorrheic adolescent females were defined as menstruating at irregular intervals of 36–90 days with a reported history of ≥6 menstrual cycles over the past 12 months [33]. The length of the menstrual cycle was calculated from the first day of menses to the day preceding the next menses [32,33]. In menstruating adolescent females, a blood sample was obtained during the follicular phase of the menstrual cycle, which occurs between days 7 and 11 from the onset of menstruation [6,32].

All participants and their parents were informed about the purposes, possible risks and aims of the investigation before they signed a written informed consent to participate in this investigation. The present investigation was approved by the Medical Ethics Committee of the University of Tartu, Estonia, and is in accordance with the ethical standards of the Declaration of Helsinki. An observational cross-sectional examination was conducted, and the current study included anthropometry, body composition, bone mineral and resting energy expenditure (REE) measurements in addition to blood analyses.

### 2.2. Bone Mineral Density and Body Composition Assessment

Body height was measured to the nearest 0.1 cm using Martin’s metal anthropometer (GPM Anthropological Instruments, Zurich, Switzerland), while body mass was measured to the nearest 0.05 kg using a medical electronic scale (A&D Instruments Ltd., Abingdon, UK) and body mass index (BMI; kg/m^2^) was calculated. In addition, the whole-body (WB) and lumbar spine (LS) areal bone mineral density (aBMD; g/cm^2^), and WB bone mineral content (BMC; kg) were measured by dual-energy X-ray absorptiometry (DXA) using the DPX-IQ densitometer (Lunar Corporation, Madison, WI, USA) equipped with proprietary software, version 3.6 [7]. The whole-body fat mass (FM), lean body mass (LBM) and WB fat percent (body fat%) were also determined by DXA [7]. Studied female adolescents were scanned while wearing light clothing and lying flat on their backs with arms at their sides. The medium scan mode and standard subject positioning were used for WB measurements and were analyzed using the extended analysis option [7]. DXA measurements and results were evaluated by the same examiner and the coefficients of variations (CVs) for the obtained results were less than 2% [7].

### 2.3. Resting Energy Expenditure Assessment

The studied female adolescents were requested to attend the laboratory after overnight fasting for at least 7 h. In addition, they were advised to use some form of non-physically demanding transportation such as cars or buses to minimize metabolic increases. To measure resting energy expenditure (REE), participants were asked to remain still and relaxed in a lying position for 15 min while breathing normally [34]. This was followed by the measurement of oxygen consumption (VO_2_) and carbon dioxide (VCO_2_) production for a 30 min period. For this purpose, a portable open-circuit spirometry system (MetaMax 3B, Cortex Biophysic GmbH, Leipzig, Germany) was used [34]. For the assessment of REE, the first and last 5 min of the measurement were deleted in order to obtain an adequate measurement [34]. The registered data were stored in 10 s intervals, while the mean of the full 20 min was used to calculate the REE of the participants [34]. The following Weir’s [35] equation was used for the calculation of REE in the studied adolescent females: Basal metabolic rate (BMR) (kcal/min) = 3.9 [VO_2_ (L/min)] + 1.1 [VCO_2_ (L/min)], and REE (kcal/day) = BMR × 1440 min [34].

### 2.4. Blood Analysis

Blood samples were obtained from the vein before breakfast and after an overnight fast, at a time between 8:00 and 9:00 a.m., with the adolescent females sitting in an upright position. The blood serum was separated and frozen at −80 °C for further analysis. Follistatin was measured using a commercially available enzyme-linked immunosorbent assay (ELISA) kit (R&D Systems Inc., Minneapolis, MN, USA). This assay had intra- and inter-assay CVs of 3.0% and 10%, respectively, and the least detection limit was 29 pg/mL. Leptin and insulin were assessed by Evidence^®^ Biochip Technology (Randox Laboratories Ltd., Crumlin, UK). This assay had intra- and inter-assay CVs of <5%. Glucose was assessed with a commercial kit (Boehringer, Mannheim, Germany). The insulin resistance index was calculated using the homeostasis model assessment (HOMA-IR): fasting insulin (µIU/mL) × fasting glucose (mmol/L)/22.5 [36].

### 2.5. Statistical Analysis

All statistical analyses were performed with the SPSS software version 21.0 package for Windows (Chicago, IL, USA). Standard statistical methods were used to calculate means and standard deviations (±SDs). All variables were checked for normal distribution using the Kolmogorov–Smirnov statistical method, and variables that were not normally distributed were log-transformed to approximate a normal distribution before the analyses. Pearson correlation coefficients were calculated to explore the bivariate relationships. In addition, partial correlation analysis was performed to assess the relationships of follistatin concentrations with bone mineral, blood biochemical and REE variables after controlling for body FM and LBM [34,37]. Multiple regression analysis was performed to identify the effects of follistatin, leptin, insulin, HOMA-IR, FM, LBM, REE and age on measured aBMD and BMC indices [7]. A *p*-value of less than 0.05 was considered significant for all analyses.

## 3. Results

The participants were 89 postmenarcheal adolescent females with different physical activity patterns and training statuses. Their mean age was 16.2 ± 1.3 years, ranging from 14 to 18 years, and all of them were at least 2 years beyond menarche. The mean BMI was 20.6 ± 1.9 kg/m^2^ and ranged from 16.5 to 24.9 kg/m^2^, demonstrating that all of the studied adolescent females had a normal body mass for their age (Table 1). All studied adolescent females were participants of different organized physical activities with different participation statuses, ranging from recreational physical activities to highly competitive athletic activities. It appeared that the mean weekly training activity was 12.2 ± 8.1 h/week. About 23% of the adolescent females represented different recreational physical activities and 77% represented different competitive athletic activities.

**Figure 1:**
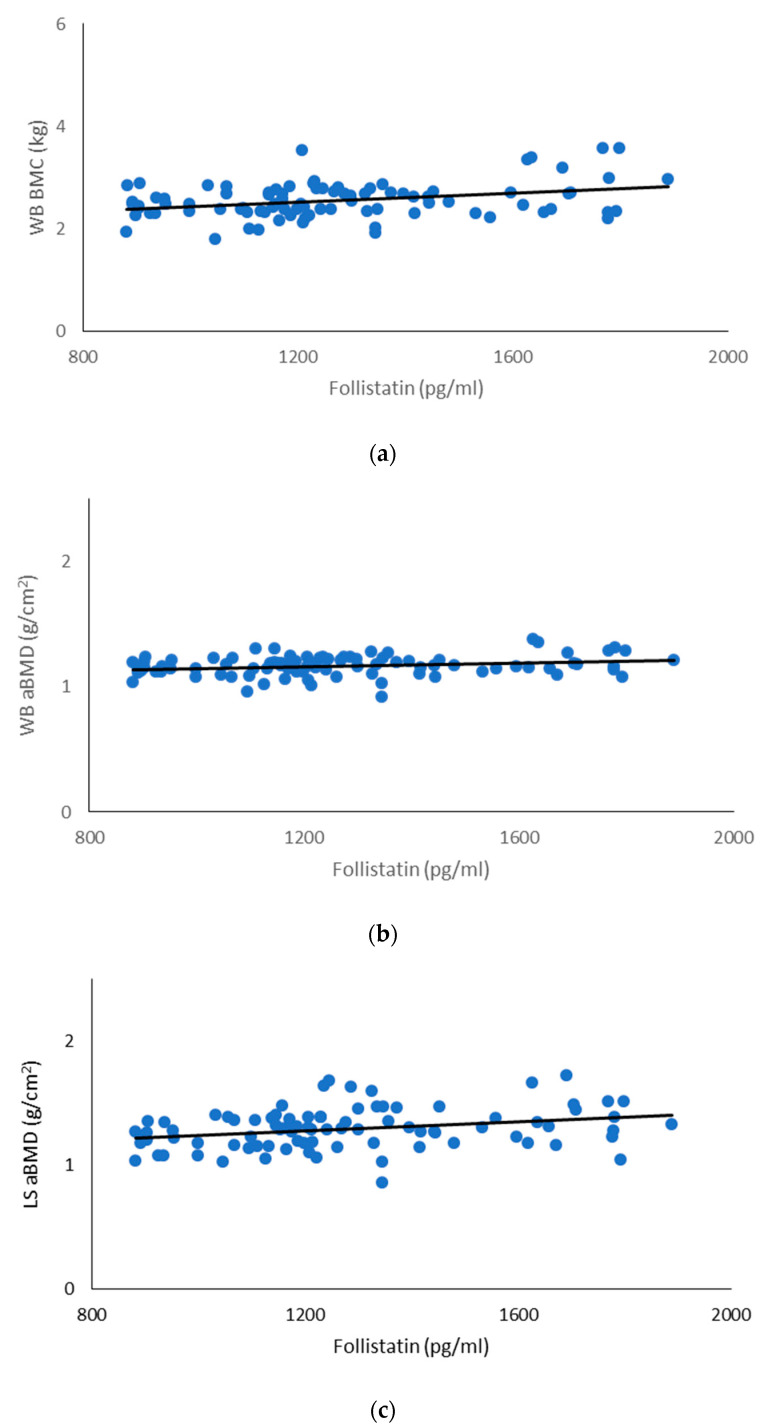
Relationships of serum follistatin levels with whole-body (WB) bone mineral content (BMC) (**a**) (r = 0.33; *p* < 0.05), WB areal bone mineral density (aBMD) (**b**) (r = 0.23; *p* < 0.05) and lumbar spine (LS) aBMD (**c**) (r = 0.29; *p* < 0.05) values.

The mean serum follistatin concentration was 1275.1 ± 263.1 pg/mL, and a bivariate correlation analysis demonstrated that the serum follistatin concentration was significantly related to the FM, LBM, WB BMC, WB aBMD, LS aBMD and REE values (Table 2; Figure 1). Partial correlation analysis showed that the serum follistatin concentration remained significantly related to LS aBMD (r = 0.21; *p* = 0.049) and REE (r = 0.21; *p* = 0.047) when adjusting for body FM and LBM values.

The multiple regression analysis models fitted to data for WB BMC, WB aBMD and LS aBMD are demonstrated in Table 3. Several independent variables including follistatin, leptin, insulin, HOMA-IR, FM, LBM, REE and age were entered in the regression analysis models. In the model where LS aBMD was the dependent variable, the independent variables that were significantly associated with LS aBMD values were LBM and follistatin, explaining 15% (R^2^ × 100; *p* < 0.0001) of the total variance in LS aBMD values. Among them, the strongest predictor was LBM (12%; *p* = 0.005), followed by follistatin (by 3%; *p* = 0.049). The strongest predictor for the WB BMC value was LBM (22%; *p* < 0.0001), followed by body FM (18%; *p* < 0.0001), and they together explained 40% (*p* < 0.0001) of the total variance in WB BMC values. Similarly, the strongest predictor for the WB aBMD value was LBM (12%; *p* < 0.0001), followed by body FM (4%; *p* = 0.027), and they together explained 16% (*p* < 0.0001) of the total variance in WB aBMD values (Table 3).

## 4. Discussion

In a relatively homogeneous group of lean adolescent females with chronically increased energy expenditure levels, positive correlations between the serum follistatin concentration and measured bone mineral values were observed. Therefore, the association between the serum follistatin level and LS aBMD value was independent of the influences of whole body FM and LBM values, and follistatin was a significant predictor of LS aBMD. However, the independent contribution of follistatin to the interindividual variance in the measured LS aBMD value was only modest. The findings of the present investigation show that follistatin appears to exert an independent effect on the LS aBMD site of the skeleton, which predominantly consists of trabecular bone in a homogeneous group of healthy adolescent females, and follistatin may represent a link between muscle tissue and trabecular bone.

The muscle–bone axis is emerging as an endocrine domain critical in the reciprocal regulation of energy and skeletal homeostasis [11,38]. This is also exemplified by circulating follistatin, a myokine that induces far-reaching effects in muscle and bone tissues throughout the lifespan [22,23,39,40]. Specifically, follistatin is regarded as promoting muscle growth through satellite cell proliferation and the inhibition of both myostatin and activin [25]. Similarly, follistatin can modulate bone metabolism by suppressing myostatin and activin signaling in bone cells [30,38]. Follistatin has been reported to stimulate osteoblastogenesis [21] and increase bone mass and density [30,31], and overexpression of follistatin leads to a greater bone mass [41]. In accordance with the predictive positive associations between follistatin and bone mineral values based on animal findings [11,30,31], we observed robust positive relationships of serum follistatin concentrations with measured BMC and aBMD indices in a homogeneous group of lean adolescent females with chronically elevated energy expenditure levels. However, we should note that these relationships were not reported in other investigations in young patients with anorexia nervosa [10] and middle-aged women with obesity [9], with both studies representing conditions of extreme changes in energy homeostasis. In contrast, a recent study demonstrated that higher circulating follistatin concentrations in women with postmenopausal osteoporosis compared with a healthy premenopausal group were negatively correlated with measured aBMD values [22]. Therefore, higher follistatin concentrations in women with postmenopausal osteoporosis were thought to be compensatory in order to restrain bone loss [22]. Accordingly, it was thought that follistatin could be related to osteoporosis in postmenopausal women and that it should be investigated further to find out whether follistatin could be used as a therapeutic target for low aBMD [22]. However, similarly with our results, circulating follistatin concentrations were found to be positively related to aBMD values in a healthy group of physically active lean young women with normal menstrual cycles [10]. It appears that data regarding follistatin effects on bone tissue in women at a specific age, stage of menstruation, bone mineral density, body composition and physical activity status are relatively scarce and contradictory, and further investigations are needed to better understand the specific role and possible mechanism(s) of follistatin in the regulation of bone homeostasis in different human populations before any conclusions can be drawn.

An interesting finding of the current study was an independent effect of serum follistatin concentration on LS aBMD, suggesting that circulating follistatin concentrations may more closely influence trabecular bone homeostasis, as LS aBMD predominantly consists of trabecular bone [22]. Similarly, the associations of the activin-to-follistatin ratios were high for LS aBMD, which predominantly consists of trabecular bone; were weakened for femoral neck aBMD, which consists of both trabecular and cortical bone; and did not exist for total hip aBMD, which predominantly consists of cortical bone [22]. In accordance, it has been shown that increased follistatin levels were associated with an increased trabecular bone volume in mice [30,31]. It has been hypothesized that the closer association of the circulating follistatin level with trabecular bone could be partially related to the higher bone turnover rate in regard to trabecular bone compared to cortical bone [22]. Furthermore, trabecular bone, including that associated with LS aBMD, is more influenced by increased energy expenditure together with increased myokine concentrations caused by chronically increased physical activity [2,11]. Circulating follistatin as a myokine, which was also correlated with the REE value in our study, may mediate muscle mass and trabecular bone mineral metabolism in the studied lean adolescent females when under conditions of chronic increases in energy expenditure. It has also been suggested that follistatin may have a protective effect on muscle mass in young females with low estrogen, insulin-like growth factor-1 and leptin concentrations, which is caused by states of energy deprivation [22]. Taking together, the results of the current study demonstrate that circulating follistatin may serve as a novel biomarker of areal bone mineral density, at least in the LS site of the skeleton, in healthy young females with chronically increased energy expenditure levels. However, the possible use of follistatin as a biomarker of areal bone mineral density should be further investigated using studies with different design and human populations.

An important part of follistatin in glucose homeostasis has been reported in individuals with different sexes, ages and body composition variables [29], while the current study did not find any relationship between circulating follistatin concentrations and measures of insulin resistance in a specific group of lean adolescent females with chronically increased energy expenditure levels. However, serum follistatin concentrations were positively related to FM and LBM values in our studied female adolescents, which is similar to findings of previously reported individuals with different body composition variables [9,29]. It has also been reported that circulating follistatin concentrations were decreased after weight loss in addition to decreases in LBM and insulin resistance values in subjects with obesity [29], while follistatin levels were affected by chronic energy deficiency due to strenuous physical activity in women with hypothalamic amenorrhea [24]. In addition, follistatin concentrations increase in states of starvation [27], and the protective mechanism which directs calories towards the survival of the female individual is also related to the protective effect of follistatin for muscle mass in conjunction with the insulin resistance it induces [22]. In our study, serum follistatin concentrations were associated with REE independent of body FM and LBM values in physically active adolescent females. These findings together suggest that circulating follistatin levels may also reflect energy status at least in individuals with chronic changes in energy expenditure.

This study had some limitations, particularly regarding its relatively small sample size and cross-sectional design, which cannot determine the cause–causality relationships. However, the number of studied adolescent females was comparable to that in recent similar investigations in this area [9,10,22]. Another limitation is the fact that the biological maturation of adolescent females was not assessed. However, all studied females were at least 2 years beyond menarche with a mean age of 16.2 years, thus representing a rather homogeneous group of individuals regarding biological maturation. A recent study demonstrated that the age that corresponded to breast developmental stage 4 was 12.5 years in white females [42]. Similarly to our study, previous investigations have also assessed only age at menarche in adolescent females with a similar age range to study the associations between blood biochemical indices and bone mineral values [7,10]. However, further longitudinal studies are necessary to evaluate the association of serum follistatin with different bone mineral and bone turnover markers in adolescent females during growth and maturation. It is also important to investigate the underlying mechanisms that link specific myokines and bone tissue in further interventional studies. The results of our study are also limited to a specific group of white adolescents within a narrow age range and with similar body composition levels. On the other hand, we present data for a healthy population with a well-defined body composition and high level of physical activity. Moreover, to the best of our knowledge, this investigation presents new data linking circulating follistatin concentrations with trabecular bone in a specific adolescent population.

## 5. Conclusions

Circulating follistatin concentrations were associated with bone mineral indices in a homogeneous group of healthy, lean adolescent females with increased energy expenditure levels. It appears that follistatin may represent a link between muscle tissue and trabecular bone, as follistatin was an independent predictor of lumbar spine areal bone mineral density, and the lumbar spine predominantly consists of trabecular bone. Accordingly, future diagnostic accuracy studies are needed to evaluate whether follistatin may serve as a novel biomarker of areal bone mineral density in conditions of chronically elevated energy expenditure. In addition, it should be investigated further to clarify whether follistatin could be used as a therapeutic target for low areal bone mineral density [22].

## Figures and Tables

**Table 1:** Mean (±SD) of participant characteristics in study population (*n* = 89).

Variables	Mean (±SD)	Range
Age (years)	16.2 ± 1.3	14.0–18.0
Age at menarche (years)	13.0 ± 1.1	11.0–15.0
Height (cm)	167.6 ± 5.0	158.1–179.4
Body mass (kg)	58.1 ± 6.7	40.0–74.8
BMI (kg/m^2^)	20.6 ± 1.9	16.5–24.9
Body fat%	24.1 ± 6.7	11.9–34.8
Body fat mass (kg)	13.9 ± 4.3	7.1–28.3
Lean body mass (kg)	41.5 ± 4.3	31.3–52.8
REE (kcal/day)	1518 ± 203	1022–1807
WB BMC (kg)	2.5 ± 0.3	1.8–3.6
WB aBMD (g/cm^2^)	1.17 ± 0.08	0.92–1.38
LS aBMD (g/cm^2^)	1.29 ± 0.16	0.86–1.72
Leptin (ng/mL)	2.2 ± 1.8	0.9–9.9
Insulin (µIU/mL)	6.8 ± 2.8	2.2–12.7
Glucose (mmol/L)	4.6 ± 0.4	3.3–5.6
HOMA-IR	1.4 ± 0.6	0.4–2.9
Follistatin (pg/mL)	1275.1 ± 263.1	881.4–1888.2

BMI, body mass index; REE, resting energy expenditure; WB, whole body; BMC, bone mineral content; aBMD, areal bone mineral density; LS, lumbar spine; HOMA-IR, homeostasis model assessment of insulin resistance.

**Table 2:** Bivariate correlation coefficients and partial correlation coefficients, after adjustment for body fat and lean body mass, between serum follistatin concentration and body composition, bone mineral, blood biochemical and resting energy expenditure variables.

Variables	Bivariate Correlation Coefficients	Partial Correlation Coefficients
Fat mass (kg)	0.21 *	-
Lean body mass (kg)	0.29 *	-
WB BMC (kg)	0.33 *	0.13
WB aBMD (g/cm^2^)	0.23 *	0.09
LS aBMD (g/cm^2^)	0.29 *	0.21 *
Leptin (ng/mL)	0.08	0.06
Insulin (µIU/mL)	0.13	0.09
Glucose (mmol/L)	0.10	0.13
HOMA-IR	0.15	0.12
REE (kcal/day)	0.29 *	0.21 *

WB, whole body; BMC, bone mineral content; aBMD, areal bone mineral density; LS, lumbar spine; HOMA-IR, homeostasis model assessment of insulin resistance; REE, resting energy expenditure. * *p* < 0.05 is statistically significant.

**Table 3:** Predictive regression models explaining the variance in bone mineral values.

Variables ^a^	Β Coefficient ± SE	*p*-Value	Partial R^2^
WB BMC (R^2^ = 0.40; *p* < 0.0001)			
LBM	0.041 ± 0.007	<0.0001	0.22
FM	0.035 ± 0.007	<0.0001	0.18
WB aBMD (R^2^ = 0.16; *p* < 0.0001)			
LBM	0.007 ± 0.002	<0.0001	0.12
FM	0.004 ± 0.002	0.027	0.04
LS aBMD (R^2^ = 0.15; *p* < 0.0001)			
LBM	0.011 ± 0.004	0.005	0.12
Follistatin	0.001 ± 0.001	0.049	0.03

WB, whole body; BMC, bone mineral content; aBMD, areal bone mineral density; LS, lumbar spine; LBM, lean body mass; FM, body fat mass. ^a^ Variables tested in the model: follistatin, leptin, insulin, HOMA-IR, FM, LBM, resting energy expenditure and age.

## Data Availability

The datasets used in this study are available from the corresponding author upon reasonable request.
